# The Importance of Undergraduate Research Experiences

**DOI:** 10.1523/ENEURO.0217-24.2024

**Published:** 2024-07-19

**Authors:** Peyton Green, Ashley Smith, Sarah M. Misemer, Jennifer N. Dulin

**Affiliations:** ^1^Department of Biology, Texas A&M University, College Station, Texas 77843; ^2^School of Performance, Visualization & Fine Arts, Texas A&M University, College Station, Texas 77843; ^3^Texas A&M Institute for Neuroscience, Texas A&M University, College Station, Texas 77843

**Keywords:** Undergraduate research

## Introduction

We are fortunate to belong to a large undergraduate R1 research institution [at the time of writing, over 57,000 undergraduate students are enrolled at Texas A&M University (TAMU)]. Research is a major part of the undergraduate experience, and in fact many of our degree plans require undergraduate students to participate in at least one semester of laboratory research. As the principal investigator (PI) of a neuroscience laboratory (J.N.D.), former Associate Director for Undergraduate Research (S.M.M.), and current undergraduate researchers (P.G. and A.S.), we each have unique perspectives on the benefits of undergraduate research experiences (UREs).

According to a 2007 report based on self-reported outcomes among undergraduates and their URE mentors, UREs increase interest in science, technology, engineering, and math (STEM) careers for over two-thirds of students who participate, and the large majority (88%) of students improve their understanding of how to conduct research (Russell et al., 2007). In another study based on self-reported outcomes, 91% of students reported that their URE solidified or increased their interest in postgraduate education (Lopatto, 2004). This study found that UREs increase interest in critical skills such as learning to work independently, tolerating obstacles, and understanding how knowledge is constructed (Lopatto, 2004). These types of tangible benefits are clear and compelling, underscoring enthusiasm for participation in UREs. However, to date most research on this topic has relied on self-reported outcomes rather than objective measures of success, underscoring a need for more quantitative research about the benefits of UREs. In today's educational climate, when many undergraduate students are recovering from educational and social setbacks in the postpandemic era, hands-on research experiences seem more critical than ever for bolstering high-level critical thinking skills and teamwork skills. Here, we discuss our own experiences with UREs and undergraduate mentorship and highlight ways to promote and enrich UREs. Each of us has contributed sections below to share our unique experiences.

## Benefits of laboratory research: the undergraduate perspective

We got involved in research as a rising senior in high school (P.G.) and a sophomore in college (A.S.). Undergraduate research presented the perfect opportunity to pursue our interests while also providing an outlet for professional development. Since we were children, we have both been dedicated to making the world a better place. Joining a research program with a focus on neuroscience presented an ideal opportunity for us to extend our education beyond the classroom while working toward developing better therapeutics for spinal cord injury, our chosen field of study. We both joined the same research lab, working on projects funded by our PI's grants as well as generous undergraduate research fellowships through Texas A&M (P.G. is currently supported by the Texas A&M College of Arts & Sciences Undergraduate Research Program). Under the direction of our PI and our graduate student and postdoc mentors, we have worked on various research projects in the wet lab, learning diverse skills including animal behavioral assessments, tissue dissection and histology, fluorescence microscopy, and image analysis. We have also participated in literature reviews and systematic analysis of research funding, which has allowed us to understand research at a higher level than is typical for undergraduates. This type of structured research environment, with clearly defined goals that are aligned with the goals of research grants, provides a high level of motivation for us students to meet milestones and get invested in real science.

There are many things that are appealing about participating in UREs. For example, undergraduate research is appealing because it fosters creativity and collaboration in the laboratory. Through this, we have learned that everyone's contribution regardless of rank is vital to the outcome of the project; while one person's knowledge and skill level may be deeper than another's, individual efforts make up the whole. Undergraduate research has provided us with the tools to communicate effectively and think critically in diverse settings. Another major benefit of UREs is the thrill of discovery—the ability to be the first person ever to answer a research question is incredibly exciting! Not only is the opportunity for discovery appealing, but the hands-on experience we have gained in the lab is invaluable, and the techniques we have learned can be applied to future projects, in college and beyond. Undergraduate research allows us to utilize state-of-the-art facilities and work alongside graduate students who will be future leaders in their fields. We have also found that UREs have taught us important life skills that would not have been gained through a typical classroom experience. For a typical undergraduate, we are normally the ones having the questions asked to us rather than us asking the questions. UREs flip the script by letting us be the ones to ask “Why?” and “How?”, taking our education into our own hands.

Participating in UREs has positively shaped our career trajectories by showing us the importance of preclinical research development and giving insight as to how therapies are translated toward healthcare settings. Many undergraduates participating in UREs aspire to go into healthcare careers. As aspiring physicians, students should have an understanding of not only the treatments being administered to patients but also the research that goes into the development of clinical therapies. Therapies are first developed on the lab bench, and without research discoveries, the advancements in healthcare would be far less consequential. Some of us started college thinking that we wanted to attend medical school after graduation, but through participation in UREs, we have pivoted toward a career in research. For example, I (P.G.) decided that because of my experience exploring the science behind therapeutic development, I would be better suited pursuing a Ph.D. in neuroscience than attending medical school. My ultimate goal is to work toward translating the work we have been doing in the lab toward a clinical treatment for people with spinal cord injury.

Before joining an undergraduate research program, there are a few things that we think students should know. We would encourage all future undergraduate researchers to identify their interests and find a lab that will provide the tools necessary to investigate those interests. In a large undergraduate institution, spaces for undergraduates in the lab can be limited, so keep an open mind when reaching out to potential advisors. After you land a position, chances are the research you will be a part of is someone's life work, so you should approach your project very seriously. Be accountable, and be honest about the amount of time that you are willing to commit to the lab—do not overextend yourself. Finally, it is important to emphasize that participating in undergraduate research is not just about performing experiments; it is about developing a scientific mindset, building relationships, and discovering your passions.

## Undergraduates enhance the quality of research

I (J.N.D.) first became involved in research during my freshman year of college, and it irrevocably shaped my career trajectory. I entered college as a freshman thinking that I wanted to go to medical school because I liked science classes, made good grades, and wanted to help people. My degree plan required one semester of laboratory research, so I joined a lab researching the biophysics of protein folding. I instantly fell in love with the mystery and uncertainty of scientific research, and over the course of 3 years of working in the lab, I decided that I wanted to become a scientist instead of a physician. Twenty-two years later, I am now a faculty member at the same university where I first fell in love with research. I have mentored over 40 undergraduates in my own research lab and won multiple awards for mentoring and teaching undergraduates. Through these experiences, I have gained a deep conviction that the quality of research is dramatically enhanced by involving undergraduates in the scientific process.

Undergrads in my lab can make substantial contributions. They work hands-on with samples, cryosectioning the brain tissue or performing immunohistochemistry. They run PCR and in situ hybridization, analyze fluorescence micrographs, and quantify animal behavioral data. Several have joined my lab as freshmen and published their own first-author papers by graduation. Their concrete contributions to the advancement of our research program cannot be understated. However, there is also a more subtle contribution of URE to the culture of the lab that is important to emphasize. In a lab full of undergraduates working alongside graduate students and postdocs, there emerges an environment where hierarchy and seniority are not necessarily the driving forces that guide interactions. Rather, all students are given a degree of responsibility that is commensurate with their unique time commitment, maturity, and dedication to their work. Top-performing undergraduates may train new grad students or lead teams in which they delegate tasks to graduate students and postdocs. In turn, graduate students gain the opportunity to mentor teams of undergrads, which prepares them to manage trainees in their postdocs and future careers. This builds a strong positive culture of teamwork and equity, instilling confidence in students as critical leadership skills are strengthened.

As the PI of a laboratory, it is my responsibility to make sure that I provide undergraduate researchers with mentorship and guidance to help them meet their goals both in and out of the laboratory. I usually meet with students one-on-one once they get settled in to ask them, “What are your goals for your future career?” If they want to go to medical school, I coach them on the importance of serving in impactful leadership positions and dedicating themselves toward healthcare-related service opportunities. If they want to pursue science as a career, we work toward building a competitive CV so that they can matriculate to their top choices of graduate program. Above all, I encourage them to think critically and independently, showing them how the scientific method works and challenging them to design experiments themselves. Undergraduate researchers are not just “hands” to generate data—they are the next generation of scientists, clinicians, and STEM professionals, and my job is to give them the best possible opportunity for success.

## Perspectives from university research program administration

I (S.M.M.) served in the capacity of Associate Director for LAUNCH: Undergraduate Research (UGR), where I directed university-level programs and policies for TAMU between 2016 and 2022. Undergraduate research is transformative for students, and it is one of the oldest and most recognized high-impact practices with myriad benefits. High-impact practices improve the student experience and carry over into employment as well as postbaccalaureate opportunities and future success. However, what is not as widely discussed is the way in which undergraduate research can have impact at the institutional level to remove barriers to access for students and faculty. During my time in the position, I led initiatives to improve the summer UREs, create discipline-specific templates for theses, and establish pipelines for funded research so we could recruit a wide variety of students and broaden participation in undergraduate research. We know undergraduate research alchemizes the student experience to produce improved grades, retention, confidence, and a sense of belonging. I believe access to that kind of education should be open to everyone.

Some of the improvements we enacted to facilitate access to summer undergraduate research started with the elimination of economic barriers and the addition of resources to encourage student applications from a wide audience. The removal of fees such as the general deposit fee for nondegree-seeking students was critical. We then tackled the creation of a mechanism to enroll students in a summer course and provide a tuition waiver to allow them access to campus services (advising, career center, bus service, student recreation center, and health center, among others). We later collaborated with Residence Life to find solutions to address food and food insecurity issues related to cultural norms, transportation, and income (community kitchens, reduced rates for refrigerator/microwave combos, recommendations to programs to include food stipends) and negotiated lower housing fees. Finally, we worked with existing programs to develop inclusive recruitment processes and applications and free access to training and development (General Lab Safety, Radiological Safety, Title IX, and Development Seminar and free poster session). Our office also provided free access to Qualtrics for PIs to customize and manage their applications, and we worked with one of our successful National Science Foundation (NSF)-funded Research Experiences for Undergraduates (REU) programs (Costa Rica) to provide workshops for PIs on how to leverage partially collected data from the applications to extend greater outreach and recruitment practices for applicants in underrepresented groups. Some of these practices included reaching out to candidates and mentors when applications were not completed to answer questions and encourage them to finish. Data collected also allowed PIs to concentrate recruitment in different geographic zones and institutions of higher learning that included 2 year community colleges (Kotinek et al., 2019). Efforts such as these allow PIs to recruit students from diverse backgrounds, improve efforts at graduate student recruitment, and fulfill NSF-REU guidelines that require diversity enhancement.

Other activities that improved students’ experiences and encouraged more participation across a variety of fields and disciplines included creation of new thesis templates and funded programs (Kotinek et al., 2019). We immediately added a humanities template to accompany the one existing STEM format [Introduction, Methods, Results, and Discussion (IMRaD)] and created a focus group comprised of scholars and practitioners in creative fields across campus to design a new creative work thesis template and learning outcomes informed by scholarly inquiry (implemented in Fall 2017). In 2019, we developed a team-based template in collaboration with the Caruth School of Dental Hygiene at TAMU Dallas. In 2020, the LAUNCH: UGR office created and released two journal-style thesis templates (one is customizable for arts, humanities, social sciences, and creative fields, and one follows the IMRaD structure) for students who were affected by constraints from the pandemic and the sudden switch to online learning. The journal-style templates have the additional benefit of alleviating economic burdens for those unable to travel to conduct research, and they eliminate physical barriers for in-person research for those with disabilities or health issues. Initiatives that evolved between 2016 and 2021 under my leadership allowed additional students to produce undergraduate research theses that accurately follow disciplinary norms in a wide variety of fields (from 2016 to 2022: 165 arts and humanities theses, 84 creative works theses, 18 journal-style theses, 20 dental hygiene theses). The creation of new templates (available at LAUNCH - Texas A&M University, 2023b) has diversified opportunities and increased participation by students in research disciplines not previously served by our Undergraduate Research Scholars (URS) thesis program, especially by students in arts, humanities, and creative fields.

Additionally, we funded opportunities to sustain teaching and undergraduate research in arts, humanities, and creative fields. In LAUNCH: UGR, I wanted to build off the success of a program I created at the Glasscock Center for Humanities Research (GCHR; Associate Director 2011–2016) for a funded 10 week summer undergraduate experience (both faculty and students receive stipends). The program consisted of a hybrid learning environment that combined a 2 week/40 h/three-credit seminar led by tenured faculty, an 8 week writing studio led by a dedicated staff member from the University Writing Center, and a public presentation of students’ work hosted by the GCHR in early fall. To create the new program, I joined with colleagues in the departments of English and Visualization, along with the Writing Center in 2018, to establish a summer gateway into the year-long URS thesis program through the Aggie Creative Collective. Over the 6 years (2016–2022), we disbursed $168,000 in funding to undergraduate students developing theses in arts, humanities, and creative fields. The funds help defray the costs associated with conducting research during the summer months and allow students to dedicate time to developing their research projects and creative artifacts. These programs also fund teaching by their faculty mentors. We also allocated over $140,000 to faculty to support summer initiative because we recognize there are far fewer grants available to faculty mentors for summer research and teaching in the arts, humanities, and creative fields.

Examples such as these illustrate how universities can leverage resources and partnerships to encourage more students to participate in transformational experiences that undergraduate research provides through hands-on experience, avenues to improve writing and presentation skills, collaborative learning, mentoring, community building, and activities that increase depth of knowledge. For more comprehensive information on undergraduate research participation and programs, please see Annual Report Archives (LAUNCH - Texas A&M University, 2023a). UREs are a pillar that makes our university, and many others, highly impactful to the professional and personal development of college students.

## Synthesis

Reviewing Editor: Christophe Bernard, INSERM &Institut de Neurosciences des Systèmes Decisions are customarily a result of the Reviewing Editor and the peer reviewers coming together and discussing their recommendations until a consensus is reached. When revisions are invited, a fact-based synthesis statement explaining their decision and outlining what is needed to prepare a revision will be listed below. The following reviewer(s) agreed to reveal their identity: NONE.

This is a nice commentary -- it is an important topic and the integration of different perspectives is useful.

A couple of comments:

* The different perspectives are a bit vague about the substance of the research experiences -- a bit more detail would be helpful: what did the individuals' URE's involve and/or what specific types of URE did Texas A&M fund/support? This could connect to some level of commentary about what *works* with a URE -- obviously just having students show up in a lab doesnt always lead to strong outcomes -- what does a student/mentor/administrator need to consider in terms of producing really *strong* UREs?

* The intro describes some of the research on the potential benefits of UREs. Can the authors make more clear which of these benefits are self-reported and which were measured objectively? That is, it seems like most current research has asked undergrads what they *felt* they gained from their UREs. It seems worth keeping track of this in part to highlight the fact that much remains to be learned about the benefits of UREs and the conditions under which they are useful.

* For the administrative components:

** Some details on how the application and recruitment process was made more equitable would be of interest.

** Some details on the degree of involvement would be really useful -- it's a large university; to what extent can another university interested in emulating these actions expect to get students involved in UREs and who is likely to particpate (demographics).

** The paragraph on templates could probably be tightened up a bit -- the stats given seem to be changes (additional 325), but some raw data would be great if available; could the templates be shared or are there resources to link to for those interested?

Finally, there might be a typo in this passage "This study found that UREs increase interest critical skills such as learning"

## Author Response

We thank the reviewers for their time and effort in reviewing our manuscript. We appreciate the positive feedback about our study and all of the productive comments that were provided. We have carefully considered all of the reviewers' comments and integrated the suggested changes into the revised version of the manuscript. Please see below for a point-by-point response to reviewers' comments (our responses are in blue). Note that the revised manuscript files have all changes highlighted.

The different perspectives are a bit vague about the substance of the research experiences -- a bit more detail would be helpful: what did the individuals' URE's involve and/or what specific types of URE did Texas A&M fund/support? This could connect to some level of commentary about what *works* with a URE -- obviously just having students show up in a lab doesnt always lead to strong outcomes -- what does a student/mentor/administrator need to consider in terms of producing really *strong* UREs? Thanks for this comment. To address, this, we have added the following to the first part of the undergraduate section: "We got involved in research as a rising senior in high school (PG) and a sophomore in college (AS). Undergraduate research presented the perfect opportunity to pursue our interests while also providing an outlet for professional development. Since we were children, we have both been dedicated to making the world a better place. Joining a research program with a focus on neuroscience presented an ideal opportunity for us to extend our education beyond the classroom while working towards developing better therapeutics for spinal cord injury, our chosen field of study. We both joined the same research lab, working on projects funded by our PI's grants as well as generous undergraduate research fellowships through Texas A&M (PG is currently supported by the Texas A&M College of Arts &Sciences Undergraduate Research Program). Under the direction of our PI and our graduate student and postdoc mentors, we have worked on various research projects in the wet lab, learning diverse skills including animal behavioral assessments, tissue dissection and histology, fluorescence microscopy and image analysis. We have also participated in literature reviews and systematic analysis of research funding, which has allowed us to understand research at a higher level than is typical for undergraduates. This type of structured research environment, with clearly defined goals that are aligned with the goals of research grants, provide a high level of motivation for we students to meet milestones and get invested in real science." The intro describes some of the research on the potential benefits of UREs. Can the authors make more clear which of these benefits are self-reported and which were measured objectively? That is, it seems like most current research has asked undergrads what they *felt* they gained from their UREs. It seems worth keeping track of this in part to highlight the fact that much remains to be learned about the benefits of UREs and the conditions under which they are useful.

This is an excellent point. We have now clarified that the studies were based on self-reported outcomes and added the following sentence to the second paragraph: "However, to date most research on this topic has relied on self-reported outcomes rather than objective measures of success, underscoring a need for more quantitative research about the benefits of UREs." For the administrative components:

Some details on how the application and recruitment process was made more equitable would be of interest.

Some details on the degree of involvement would be really useful -- it's a large university; to what extent can another university interested in emulating these actions expect to get students involved in UREs and who is likely to particpate (demographics).

Both of these comments are now addressed in the following paragraph:

Some of the improvements we enacted to facilitate access to summer undergraduate research started with the elimination of economic barriers and the addition of resources to encourage student applications from a wide audience. The removal of fees such as the general deposit fee for non-degree seeking students was critical. We then tackled the creation of a mechanism to enroll students in a summer course and provide a tuition waiver to allow them access to campus services (advising, career center, bus service, student recreation center, and health center, among others). We later collaborated with Residence Life to find solutions to address food and food insecurity issues related to cultural norms, transportation, and income (community kitchens, reduced rates for refrigerator/microwave combos, recommendations to programs to include food stipends) and negotiated lower housing fees. Finally, we worked with existing programs to develop inclusive recruitment processes and applications, and free access to training and development (General Lab Safety, Radiological Safety, Title IX, and Development Seminar and free poster session). Our office also provided free access to Qualtrics for PIs to customize and manage their applications, and we worked with one of our successful NSF- funded REU programs (Costa Rica) to provide workshops for PIs on how to leverage partially collected data from the applications to extend greater outreach and recruitment practices for applicants in underrepresented groups. Some of these practices included reaching out to candidates and mentors when applications were not completed to answer questions and encourage them to finish. Data collected also allowed PIs to concentrate recruitment in different geographic zones and institutions of higher learning that included two-year community colleges [3]. Efforts such as these allow PIs to recruit students from diverse backgrounds, improve efforts at graduate student recruitment, and fulfill NSF-REU guidelines that require diversity enhancement.

The paragraph on templates could probably be tightened up a bit -- the stats given seem to be changes (additional 325), but some raw data would be great if available; could the templates be shared or are there resources to link to for those interested? This paragraph has been shortened up. We have now provided stats on the numbers of theses published, and a link to the templates has now been included.

Finally, there might be a typo in this passage "This study found that UREs increase interest critical skills such as learning" We corrected this sentence.
